# Mitophagy regulates mitochondrial number following pharmacological induction of mitochondrial biogenesis in renal proximal tubule cells

**DOI:** 10.3389/fphar.2024.1344075

**Published:** 2024-02-05

**Authors:** Kevin A. Hurtado, Rick G. Schnellmann

**Affiliations:** ^1^ Department of Pharmacology and Toxicology, College of Pharmacy, University of Arizona, Tucson, AZ, United States; ^2^ Southern Arizona VA Health Care System, Tucson, AZ, United States; ^3^ Southwest Environmental Health Science Center, University of Arizona, Tucson, AZ, United States

**Keywords:** mitochondrial biogenesis, mitophagy, proximal tubule, autophagy, HTR1F

## Abstract

**Background:** Mitochondrial biogenesis (MB) induction through the activation of the 5-Hydroxytriptamine (5-HT) 1F receptor (HTR1F) is a promising mechanism for the treatment of diseases characterized by mitochondrial dysfunction, such as acute kidney injury (AKI). While several studies report pharmacological activation of MB in the proximal tubule, it is unclear how the proximal tubule regulates itself once the pharmacological activation is removed. Mitophagy is the process of selective mitochondria degradation. We hypothesize that mitophagy decreases mitochondrial number after pharmacological stimulation and restore mitochondrial homeostasis.

**Methods:** Renal proximal tubules were treated at time 0hr with LY344864 or vehicle for 24 h and then removed. LY344864, a selective HTR1F agonist, induces MB in renal proximal tubules as previously reported (Gibbs et al., Am J Physiol Renal Physiol, 2018, 314(2), F260–F268). Vehicle and pharmacological reagents were added at the 24 h time point. Electron microscopy was used to assess mitochondrial morphology, number, and autolysosomes. Seahorse Bioscience XF-96 extracellular flux analyzer was used to measure maximal mitochondrial oxygen consumption rates (FCCP-OCR), a functional marker of MB.

**Results:** LY344864 treatment increased FCCP-OCR, phosphorylation of protein kinase B (AKT), peroxisome proliferator-activated receptor γ coactivator-1alpha (PGC-1α), and mitochondrial number after 24 h. These endpoints decreased to baseline 24 h after LY344864 removal. Treatment with ROC-325, an autophagy inhibitor, increased Sequestosome-1 (SQSTM1/P62) and microtubule-associated protein-1 light chain 3 (LC3B) after 24 h of treatment. Also, ROC-325 treatment sustained the elevated mitochondrial number after LY344864 pre-treatment and removal.

**Conclusion:** These data revealed that inhibition of autophagy extends elevated mitochondrial number and function by preventing the lysosomal degradation of mitochondria after the removal of LY344864.

## 1 Introduction

### 1.1 Mitochondrial homeostasis and quality control mechanisms

Mitochondria are ovaloid-shaped dynamic organelles that not only produce energy, but also regulate proliferation, redox processes, lipid metabolism, and programmed cell death (Osellame et al., 2012; Ravanelli et al., 2020; Schulte et al., 2023). Mitochondrial homeostasis is tightly controlled by a complex network of quality control mechanisms that have been developed by eukaryotic cells during evolution to withstand environmental stressors (Bhargava and Schnellmann, 2017; Tang et al., 2021). The three major quality control mechanisms that maintain optimal mitochondrial function in organs like the brain, heart, and kidneys are mitochondrial biogenesis (MB), mitochondrial dynamics (fission and fusion), and mitophagy (Picca et al., 2018). MB is commonly defined as the production of new and functional mitochondria and has been reported to increase during exercise, starvation, cell division and cell differentiation (Finck and Kelly, 2006; Jornayvaz and Shulman, 2010; Lira et al., 2010; Bhargava and Schnellmann, 2017). Mitochondrial fusion is the combination of mitochondria fragments by the merging of their membranes (Adebayo et al., 2021). Mitochondrial fusion facilitates metabolite and gene product exchange between fused mitochondria and normally occurs upon greater energetic demand (Youle and van der Bliek, 2012; Adebayo et al., 2021). Mitochondrial fission refers to the fractionation of mitochondria into two distinct mitochondrial organelles and is essential for the management of mitochondria per cell during cell division (Youle and van der Bliek, 2012). Mitophagy, a selective version of autophagy for mitochondria, is a mechanism by which damaged or redundant mitochondria are eliminated by phagosomal engulfing and further degradation when fused with autolysosomes (Um and Yun, 2017; Wang et al., 2020; Ma et al., 2022). All together, these mechanisms fluctuate depending on metabolic demands to maintain mitochondrial homeostasis.

The kidney filters the blood approximately every hour and selectively reabsorbs solutes and excretes waste products (Parikh et al., 2015). Mitochondria are dense in the proximal tubule, where most of the glomerular filtrate is reabsorbed, to supply energy for transporters. (Scholz et al., 2021). Thus, the proximal tubule is dependent on optimal functioning of mitochondrial quality control mechanisms (Tang et al., 2021). Mitochondrial dysfunction and alteration of mitochondrial quality control mechanisms has been associated with the progression of kidney injury and diseases such as acute kidney injury (AKI) and diabetic kidney disease (DKD) (Che et al., 2014; Ni et al., 2015; Parikh et al., 2015; Cleveland and Schnellmann, 2023a). Structural alterations of the mitochondria and downregulation of peroxisome proliferator-activated receptor γ coactivator-1alpha (PGC-1α), the master regulator of MB, are associated with the progression of AKI (Lynch et al., 2018). Importantly, mitophagy plays a pivotal role in renal function and renal recovery after AKI via clearance of damaged mitochondria (Ishihara et al., 2013; Tang et al., 2018; Livingston et al., 2019; Zuo et al., 2020). Mitophagy regulates MB and mitochondrial density through the degradation of damaged and redundant mitochondria (Bragoszewski et al., 2017; Um and Yun, 2017). Inversely, mitophagy is regulated indirectly by PGC-1α activation of autophagy modulators such as (SQSTM1/P62) and microtubule-associated protein-1 light chain 3 (LC3B) in murine skeletal muscle (Lira et al., 2013; Halling and Pilegaard, 2020). Despite some studies that suggest an association between MB and mitophagy, they have not been explored in the context of pharmacological induction of MB. Thus, there is considerable interest in understanding the impact pharmacological agents on mitochondrial regulation and control mechanisms that restore mitochondrial homeostasis.

Our group has pursued the induction of MB for the treatment of diseases that are characterized by mitochondrial dysfunction such as stroke, Parkinson’s disease, spinal cord injury, DKD, and AKI (Gibbs et al., 2016; Scholpa et al., 2018; Simmons et al., 2021; Hurtado et al., 2023a; Cleveland and Schnellmann, 2023b). Recent studies suggest that pharmacological activation of the 5-hydroxytriptamine (5-HT)_1F_ receptor (HTR1F) increases PGC-1α, MB and mitophagy, and accelerates recovery in an AKI mice model (Garrett et al., 2014; Hurtado et al., 2023a; Hurtado et al., 2023b). LY344864, a HTR1F agonist, increased MB signaling pathway that starts with the phosphorylation of AKT and ends in the phosphorylation of PGC-1α for its translocation into the nucleus and upregulation of mitochondrial transcription factors such as mitochondrial transcription factor A (Gibbs et al., 2018; Chen et al., 2022). Collectively, these studies show that activation of these receptors increased MB, rescue organ function, and restores mitochondrial homeostasis to baseline.

While the signaling pathways for MB have been studied, little is known about the mechanism that restores MB back to baseline. We hypothesized that mitophagy plays an important role in restoring mitochondrial homeostasis after pharmacological induction of MB in the renal proximal tubule.

## 2 Methods

### 2.1 Reagents

LY344864 was purchased from Tocris (Ellsville, MO, Cat# 24-511-0). ROC-325 was purchased from Selleck Chemical (Houston, TX, Cat# 1859141).

### 2.2 Isolation and culture of RPTCs

Female New Zealand White rabbits (1.8–2 kg) were purchased from Charles River (Oakwood, MI). RPTCs were isolated using the iron oxide perfusion method and grown in 35-mm tissue culture dishes under improved culture conditions similar to what is observed *in vivo* (Nowak and Schnellmann, 1995). The culture medium was a 1:1 mixture of DMEM-F-12 (without glucose, phenol red, or sodium pyruvate) supplemented with 15 mM HEPES buffer, 2.5 mM L-glutamine, 1 μM pyroxidine HCl, 15 mM sodium bicarbonate, and 6 mM lactate. Hydrocortisone (50 nM), selenium (5 ng/mL), human transferrin (5 μg/mL), bovine insulin (10 nM), and l-ascorbic acid-2-phosphate (50 μM) were added to fresh culture medium. Confluent RPTCs were used for all experiments as previously reported (Nowak and Schnellmann, 1995). All animal experiments were approved by the Institutional Animal Care and Use Committee at the University of Arizona.

### 2.3 Analysis of oxygen consumption

RPTC were plated and cultured in 96-well respiratory plates. 18,000 cells were seeded per well. Experiments were conducted on the fourth or sixth day after planting when cells had formed a confluent monolayer. The oxygen consumption rate (OCR) of RPTCs was measured using the Seahorse Bioscience XF-96 Extracellular Flux Analyzer. Each 96-well assay plate was treated with vehicle (DMSO; <0.5%) or LY344864 at 10 nM as reported (Gibbs et al., 2018). Basal OCR was measured before injection of carbonyl cyanide 4-(trifluoromethoxy) phenylhydrazone (FCCP; 2 μM) to measure the uncoupled OCR (FCCP-OCR), a marker of MB (Beeson et al., 2010).

### 2.4 Transmission electron microscopy (TEM) analysis

Kidney cortex samples were fixed in 2.5% glutaraldehyde and then PBS (Thermo-Fisher Scientific; Waltham, MA; Cat# 50-366-997 & 13-151-014), stored overnight, and submitted to the TEM core facility at the University of Arizona. Images were obtained with a FEI Tecnai Spirit Transmission Electron Microscope (Hillsboro, OR) at 100 kV. TIFF images (8-bit) were captured with an XR41 CCD digital camera (Woburn, MA) at 6,000×. For all cases, 5–6 images were analyzed per sample and mitochondrial morphology per field were calculated. Each image represents 1-2 cells per field. Each field contained ∼30-80 mitochondria. Mitochondria length was obtained using the major axis as reported previously (Lam et al., 2021). TEM images were analyzed utilizing MathLab2020b software. Mitochondria lengths were obtained using the major axis. Autophagic vacuoles represented double membrane organelles with clear membranes and any undegraded cargo. Mitophagic vacuoles represented double membrane organelles with undegraded mitochondria exclusively as cargo.

### 2.5 Immunoblotting

Protein was extracted from kidney cortices using RIPA buffer (50 mM Tris-HCl, 150 mM NaCl, 0.1% SDS, 0.5% sodium deoxycholate, 1% Triton X-100, pH 7.4) with protease inhibitor cocktail (1:100 Millipore Sigma; Burlington, MA, Cat# P8340), 1 mM sodium fluoride, and 1 mM sodium orthovanadate (Thermo-Fisher Scientific; Waltham, MA; Cat# S299100 and AC205330500, respectively). Membranes were visualized using chemiluminescence (Thermo-Fisher Scientific; Waltham, MA; Cat# PI34076) on a GE ImageQuant LAS4000 (GE Life Sciences; Pittsburgh, PA). Optical density was quantified with ImageJ software. Primary antibodies were purchased from Abcam (Cambridge, MA): PGC-1α (1:1000, Cat # ab191838). NOVUS Biologicals (Centennia, CO): LC3B, (1:1000, Cat# NB6001384). Cell Signaling Technology (Danvers, MA): DRP1 (1:1,000, Cat#5391S), P62 (1:1,000, Cat#5114S), Phospho-AKT (Ser473) (1:1,000, Cat#4060S). AKT (1:1,000, Cat#9262S). Santa Cruz Biotechnology: Beta-actin (1:1,000, Cat#SC-47778). Secondary antibodies: Goat Anti-Rabbit IgG H&L (HRP) (1:10,000, Abcam, Cat# ab6721), Rabbit Anti-Mouse IgG H&L (HRP) (1:10,000, Abcam, Cat# 6728), and Donkey Anti-Goat IgG H&L (HRP) (1:10,000, Abcam, Cat# ab97110) were used as secondary antibodies. All antibodies were validated for their respective targets. See their respective websites using the provided catalog numbers for a detailed description of their validation processes.

### 2.6 Statistical analysis

For experimental groups (N = 4–6), RPTC from one animal represents an *n* = 1. Statistical significance was determined by one-way ANOVA followed by a Tukey’s post-hoc test. Data were analyzed using GraphPad Prism software (La Jolla, CA) and *p* < 0.05 was considered statistically significant. Different letters represent statistical differences between groups.

## 3 Results

### 3.1 Treatment with LY344864 increases mitochondrial respiration, phosphorylation of AKT, PGC-1α and mitochondrial number in RPTC and then decreases to baseline 24 h after removal

Previously, we showed that LY344864 (10 nM) increases the MB biomarkers, FCCP-OCR, mitochondrial proteins and mitochondrial DNA in RPTC after 24 h of treatment (Gibbs et al., 2018). LY344864 initiated the MB signaling pathway by the phosphorylation of AKT in serine 473 (Gibbs et al., 2018).

**FIGURE 1:**
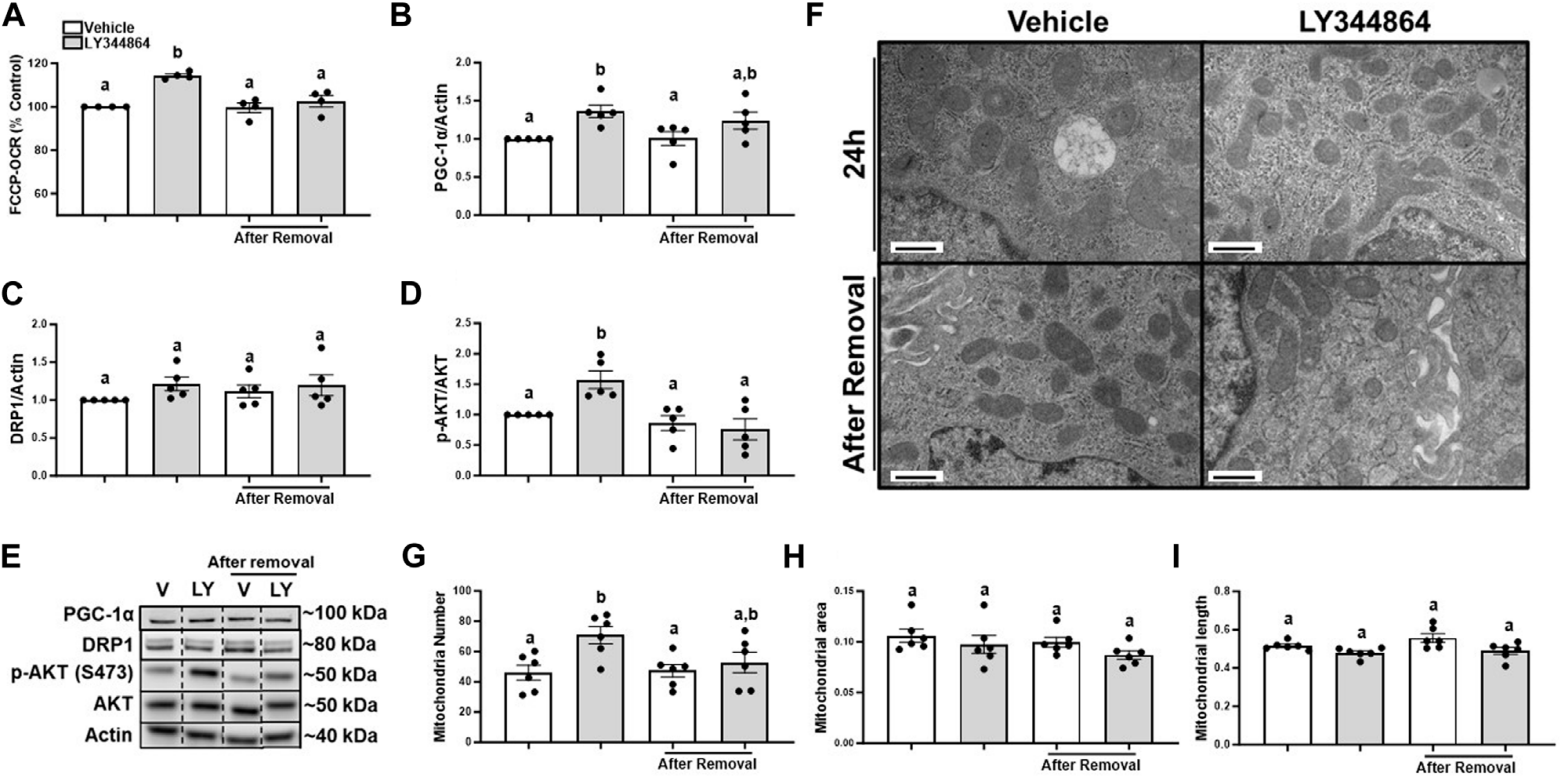
Treatment with LY344864 increases FCCP-OCR levels, phosphorylation of AKT, and mitochondrial number and returns to baseline after 24 h of removal in RPTC. **(A)** FCCP-OCR levels analysis. **(B)** Densitometry analysis of PGC-1α. **(C)** Densitometry analysis of DRP1. **(D)** Densitometry analysis of p-AKT and AKT. **(E)** Representative immunoblots. **(F)** Representative electron micrographs. **(G)** Mitochondrial number. **(H)** Mitochondrial area **(I)** Mitochondrial length. Data represents *n* = 4–6 and are expressed as mean ± SEM; *p* < 0.05 compared by a one-way ANOVA (followed by a Dunnet’s post-hoc test). Different letters on top of bars represent different statistical significance. Scale bars represent 500 nm.

It is unknown if FCCP-OCR or the signaling pathway remained elevated after the removal of LY344864. Thus, we treated RPTC with LY344864 for 24h, measured FCCP-OCR, removed LY344864 and measured FCCP-OCR again. RPTC FCCP-OCR increased 15% at 24 h and decreased to control values 24 h after removal of LY344864 (Figure 1A). PGC-1α increased 36% at 24 h and decreased to control values 24 h after removal of LY344864 (Figures 1B, E). Dynamin-related protein 1 (DRP1), master regulator or mitochondrial fission, did not change with LY344864 or after removal (Figures 1C, E). Phosphorylation of AKT increased 57% after 24 h of treatment and returned to baseline 24 h after the removal (Figures 1D, E), confirming the initiation of the signaling pathway. These data reveal that the MB signaling pathway and FCCP-OCR were activated by LY344864 and stopped by the removal.

To document mitochondria number increase, electron microscopy was performed and mitochondria number and morphology measured after treatment with LY344864 and 24 h after its removal in RPTC. Quantitative electron micrograph analysis demonstrated mitochondria number per field increased by 51% in the LY344864 group compared to vehicle group after 24 h (Figures 1F, G). However, mitochondrial number per field was restored to baseline after 24 h of removal (Figures 1F, G). Mitochondrial area and mitochondrial length were not affected by LY344864 after the first 24 h or its removal (Figures 1F, H, I). These data demonstrate that MB occurs within 24 h and recedes to control levels in 24 h. DRP1 analysis and electron microscopy analysis confirmed the decreased mitochondrial number was not due to mitochondrial fission (Figures 1C, H, I).

### 3.2 ROC-325 induces accumulation of P62 and LC3B in RPTC

**FIGURE 2:**
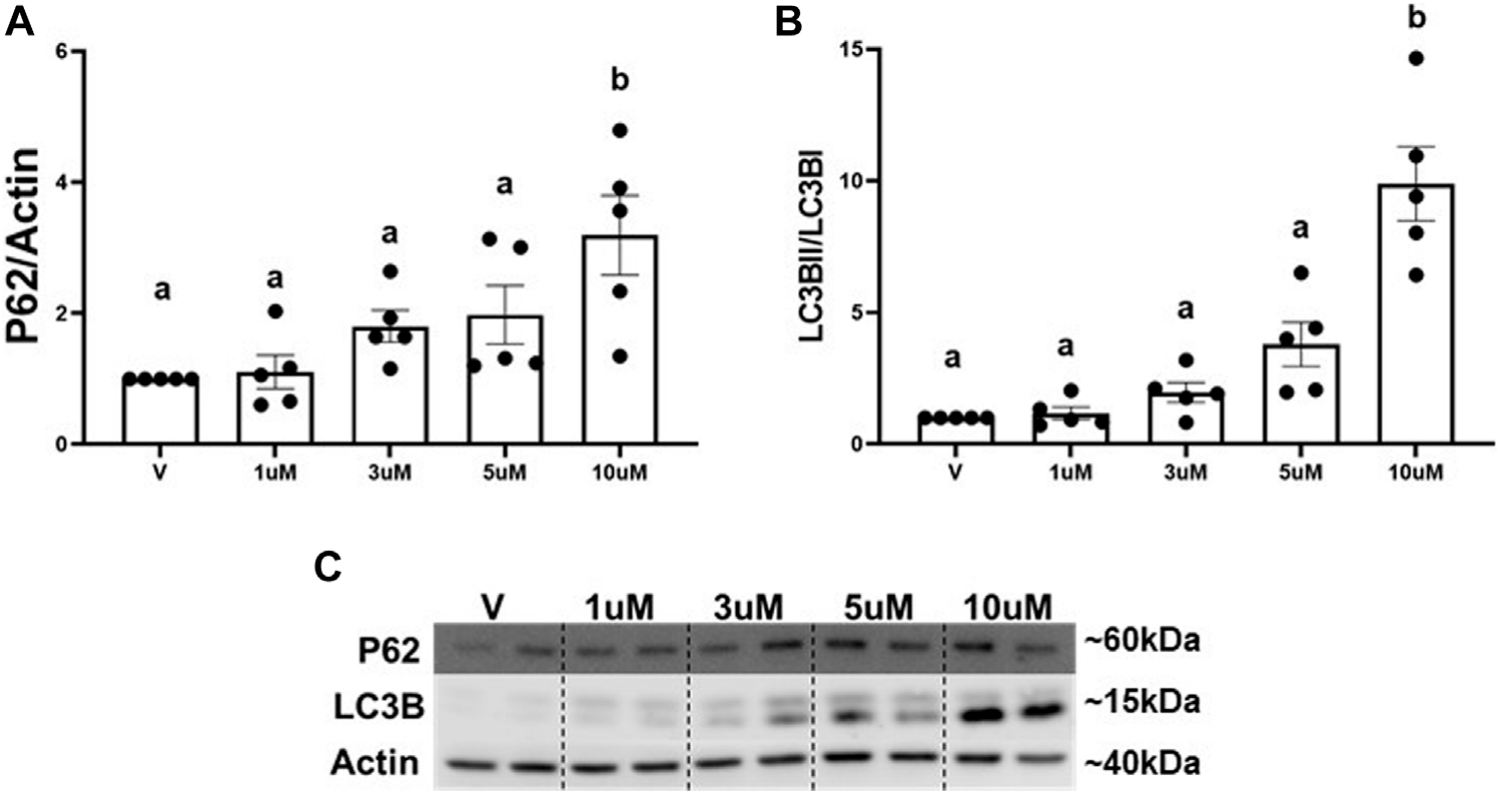
ROC-325 induces accumulation of P62 and LC3B in RPTC. **(A)** Densitometry analysis of P62. **(B)** Densitometry analysis of LC3B. **(C)** Representative immunoblots. Data represents *n* = 6 and are expressed as mean ± SEM; *p* < 0.05 compared by a one-way ANOVA (followed by a Dunnet’s post-hoc test). Different letters on top of bars represent different statistical significance.

The main mechanism attributed to the degradation of excess whole mitochondria is mitophagy (Um and Yun, 2017; Swerdlow and Wilkins, 2020). We utilized an autophagy inhibitor (ROC-325) (Carew et al., 2017) to determine if the decrease in mitochondria is regulated by mitophagy. Numerous reports demonstrate increased P62 and LC3BII, key adaptors molecules that bind to cargo (e.g., mitochondria) for autophagosomal degradation, are a result of autophagy and mitophagy inhibition (Nawrocki et al., 2019; Maestro et al., 2022). A concentration response curve for ROC-325 in RPTC was performed to identify the optimal minimal concentration to inhibit autophagy and mitophagy. Immunoblot analysis revealed that P62 and LCBII increased 3-fold and 9-fold, respectively, at 10 μM of ROC-325 when added at the 24 h time point in RPTC (Figures 2A–C). We selected 10 μM ROC-325 for all further experiments.

### 3.3 ROC-325 maintains elevated mitochondrial number after 24 h removal of LY344864 in RPTC

**FIGURE 3:**
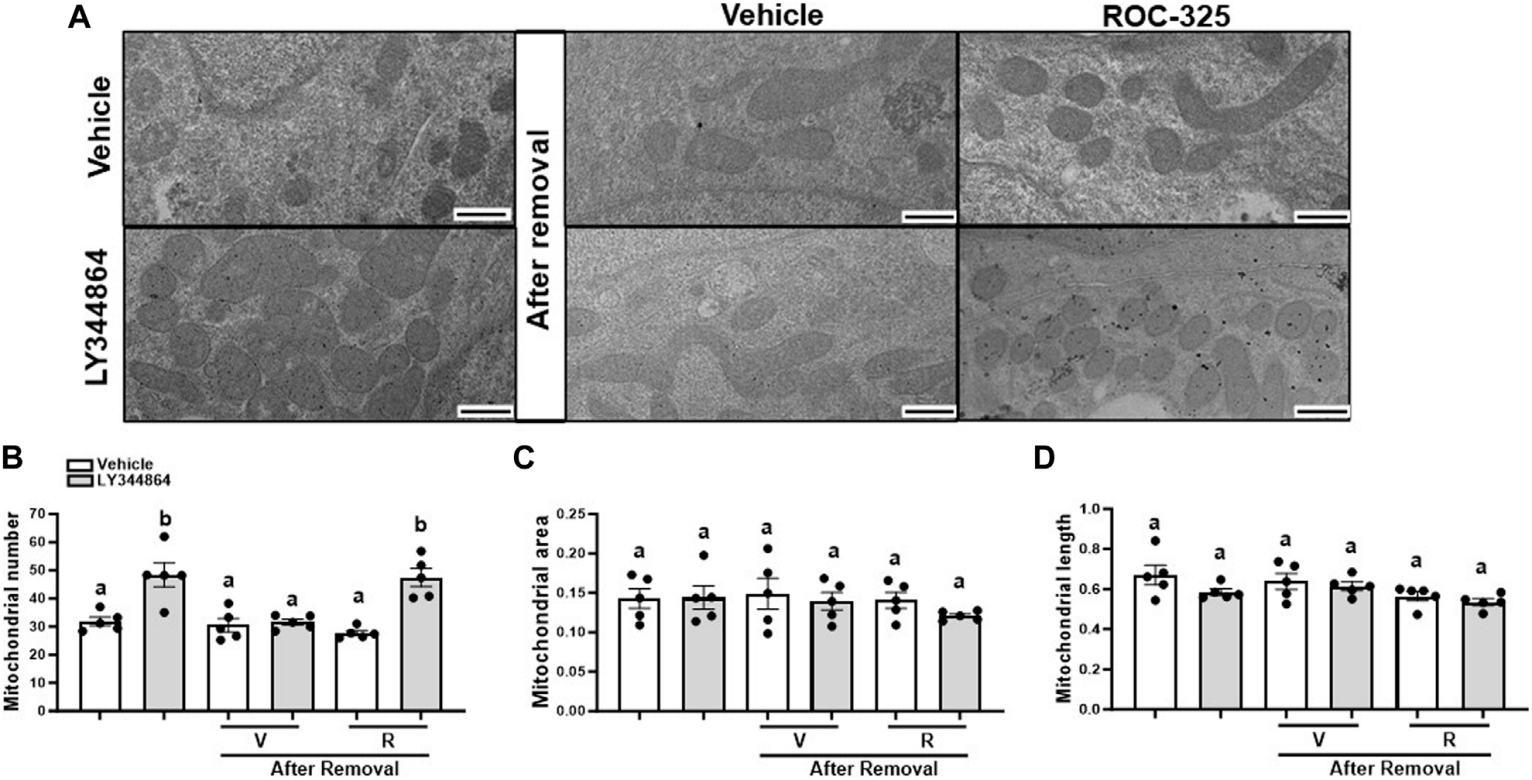
ROC-325 maintains elevated mitochondrial number after 24 h removal of LY344864 in RPTC. **(A)** Representative electron micrographs. **(B)** Mitochondrial number. **(C)** Mitochondrial area **(D)** Mitochondrial length. Data represents *n* = 5 and are expressed as mean ± SEM; *p* < 0.05 compared by a one-way ANOVA (followed by a Dunnet’s post-hoc test). Different letters on top of bars represent different statistical significance. Scale bars represent 500 nm.

Mitochondria number returns to baseline 24 h after the removal of LY344864. To explore the role of mitophagy, RPTC were pretreated with LY344864 for 24h, LY344864 was removed, and treated with ROC-325 for 24 h. Electron micrographs quantitative analysis revealed that LY344864 increased mitochondria number per field by 52% compared to vehicle (Figures 3A, B). Mitochondrial number per field (Figures 3A, B) after ROC-325 for 24 h remained elevated. All groups showed similar mitochondrial area and length, independent of treatment (Figures 3A, C, D).

### 3.4 ROC-325 increases the number of autophagic vacuoles after 24 h removal of LY344864 in RPTC

**FIGURE 4:**
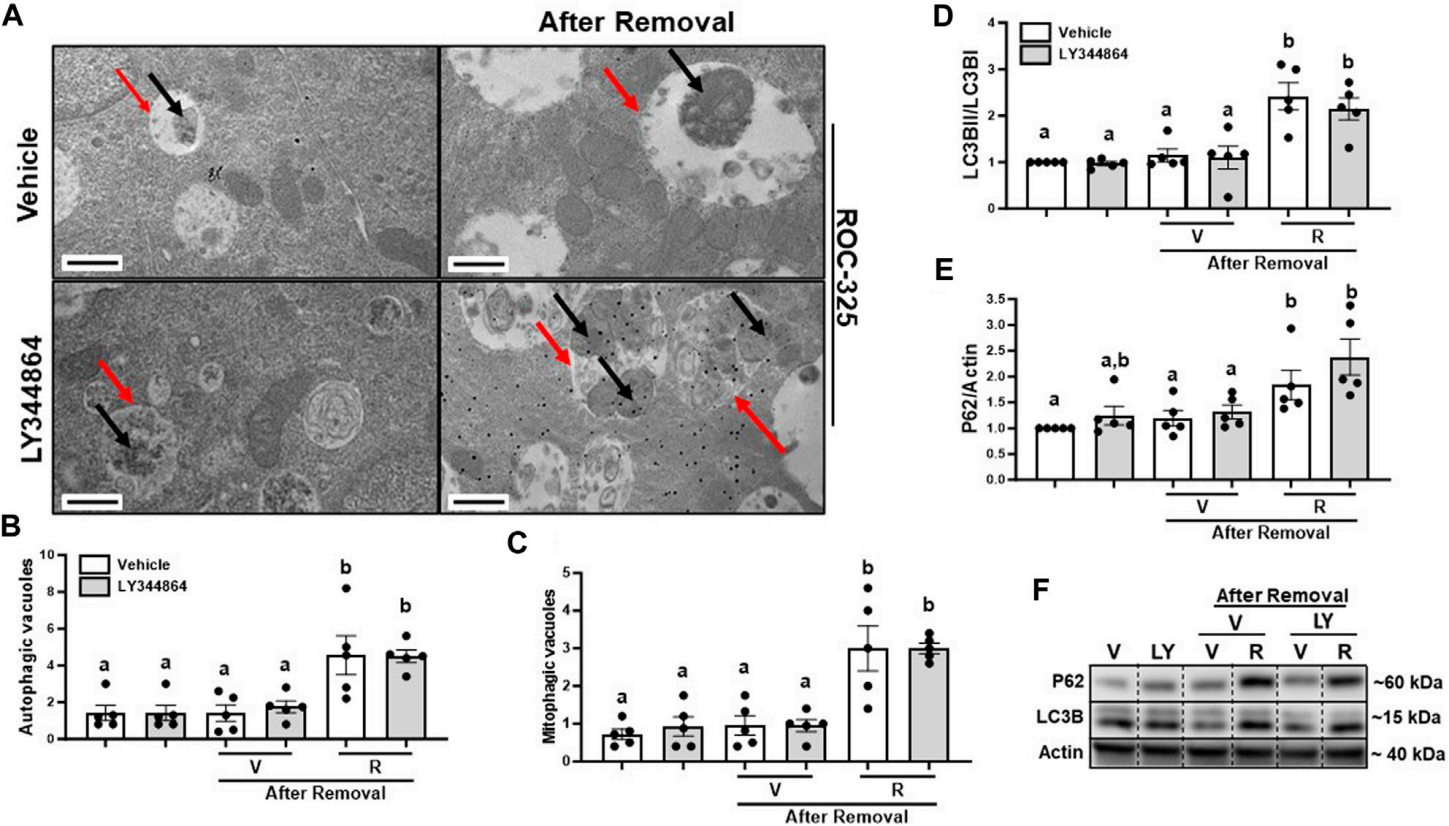
ROC-325 increases the number of autophagic and mitophagic vacuoles and accumulation of LC3Band P62 after 24 h removal of LY344864 in RPTC. **(A)** Representative electron micrographs. **(B)** Autophagic vacuoles number. **(C)** Mitophagic vacuoles number. **(D)** LC3B densitometry analysis. **(E)** P62 densitometry analysis. **(F)** Representative immunoblots. Data represents *n* = 5 and are expressed as mean ± SEM; *p* < 0.05 compared by a one-way ANOVA (followed by a Dunnet’s post-hoc test). Red arrows point at autophagic vacuole. Black arrows point at mitochondria. Different letters on top of bars represent different statistical significance. Scale bars represent 500 nm.

To determine the effect of ROC-325 on mitophagy after MB, we treated RPTC with ROC-325 for 24 h following LY344864 removal. Pharmacological treatment with ROC-325 is characterized by increased number of autophagic vacuoles with undegraded cargo (Nawrocki et al., 2019). We observed a 4-fold increase number of autophagic vacuoles, represented by double membrane organelles with undegraded cargo, after 24 h of treatment with ROC-325 (Figures 4A, B). Mitophagic vacuoles are double membrane organelles with undegraded mitochondria exclusively as cargo, also increased 4-fold with ROC-325 treatment compared to vehicle (Figures 4A, C). These results indicate ROC-325 inhibits autophagy following LY344864 removal.

As mentioned before, increased LC3BII and P62 indicate inhibition of autophagy. Immunoblot analyses were performed to study accumulation of LC3BII and P62 after 24 h treatment with ROC-325 following LY344864 removal. LC3BII increased 2-fold and P62 2.5-fold, respectively, after 24 h of treatment with ROC-325 following LY344864 removal (Figures 4D–F). Taken together, these data support the idea that autophagy inhibition is linked to persistent elevated mitochondrial number.

## 4 Discussion

MB induction can be characterized by increased mitochondrial number, mitochondrial respiration, and mitochondrial gene expression (Popov, 2020). Work from our group showed that LY344864, a HTR1F agonist, increased MB *in vitro* and *in vivo* (Garrett et al., 2014; Gibbs et al., 2018). In addition, our group reported that LY346864 treatment increased MB and accelerated recovery after spinal cord injury and AKI in mice (Garrett et al., 2014; Simmons et al., 2020). LY344864 has an 80-fold higher selectivity for the HTR1F than any other 5-HT receptor and has been shown to decrease neurogenic dural inflammation, a characteristic of neurovascular type headaches, after intravenous and oral administration in rats (Phebus et al., 1997; Goadsby and Classey, 2003; Murray et al., 2011). Ultimately, LY344864 development was ceased for toxicity.

Here, we report not only increased mitochondrial respiration and mitochondrial number per field following LY344864 treatment in RPTC, but also the duration of this pharmacological increase of MB. Expression of MB markers were restored to control levels in RPTCs 24 h after the treatment of LY344864 was removed. These results suggest that RPTC rapidly regulates mitochondria density and function to maintain mitochondrial homeostasis.

Mitochondrial quality control mechanisms are tightly regulated to preserve mitochondrial homeostasis and alterations such as decreased MB, excessive fission, and impaired mitophagy are characteristics of many reno-pathologies (Garrett et al., 2014; Bhargava and Schnellmann, 2017; Cleveland and Schnellmann, 2023a; Hurtado et al., 2023b). Pharmacologically targeting of these mitochondrial mechanisms may support kidney recovery from injury or disease (Bhargava and Schnellmann, 2017; Tang et al., 2018; Bhatia and Choi, 2019; Cote et al., 2020; Zhang et al., 2021; Cleveland and Schnellmann, 2023a). However, the crosstalk among mitochondrial quality control mechanisms to maintain mitochondrial homeostasis has not been extensively studied. The goal of this study was to elucidate the mechanism that restores mitochondrial number back to baseline after LY344864 removal.

Mitophagy is a cellular process that can regulate the mitochondrial density in a cell (Wang et al., 2020). During mitophagy, whole mitochondria are engulfed by double membrane vacuoles called autophagosomes. The vacuoles packaged with mitochondria are then fused with lysosomes and degraded (Kobayashi et al., 2020; Zuo et al., 2020). The autophagy inhibitor ROC-325 is reported to prevent the fusion of autophagosomes and lysosomes, increasing vacuoles with undegraded cargo, and increasing autophagy/mitophagy adaptor modulators P62 and LC3B *in vitro* (Nawrocki et al., 2019; Gureev et al., 2020). Our results demonstrate that ROC-325 treatment maintained mitochondria number after removing the upstream stimulus, LY344864. Furthermore, P62 and LC3B, markers of mitophagy, accumulated with ROC-325 treatment in RPTC. TEM confirmed autophagosomes with undegraded cargo (autophagic vacuoles). Also, TEM confirmed autophagosomes with undegraded mitochondria (mitophagic vacuoles). These data suggest that ROC-325 treatment decreased fusion of autophagosomes and lysosomes, resulting in decreased degradation of mitochondrial mass. Importantly, RPTC that were treated with LY344864 and then treated with ROC-325 had autophagosomes with a higher density of mitochondria compared to vehicle group (Figure 4A). These findings explain the remaining elevated mitochondria number (Figures 3A, B) This suggests that inhibition of mitophagy prevents the restoration of density of mitochondria after the removal of LY344864 in RPTC.

To our knowledge, this is the first study to investigate the role of mitophagy as a mitochondrial homeostatic mechanism following MB treatment in the kidney. While several mechanisms play a role in mitochondrial homeostasis, these findings identify mitophagy as a key regulator following MB treatment.

## Data Availability

The raw data supporting the conclusion of this article will be made available by the authors, without undue reservation.
